# Genomic Characterization of a Circovirus Associated with Fatal Hemorrhagic Enteritis in Dog, Italy

**DOI:** 10.1371/journal.pone.0105909

**Published:** 2014-08-22

**Authors:** Nicola Decaro, Vito Martella, Costantina Desario, Gianvito Lanave, Elena Circella, Alessandra Cavalli, Gabriella Elia, Michele Camero, Canio Buonavoglia

**Affiliations:** Department of Veterinary Medicine, University of Bari, Valenzano, Bari, Italy; Columbia University, United States of America

## Abstract

Dog circovirus (DogCV) was identified in an outbreak of enteritis in pups in Italy. The disease was observed in 6 young dachshunds pups of a litter from a breeding kennel and caused the death of 2 dogs. Upon full-genome analysis, the virus detected in one of the dead pups (strain Bari/411–13) was closely related to DogCVs that have been recently isolated in the USA. The present study, if corroborated by further reports, could represent a useful contribution to the knowledge of the pathogenic potential of DogCV and its association with enteritis in dogs.

## Introduction

Canine enteritis can be caused by a number of viral, bacterial or parasitic agents. The most common viral entero-pathogens are canine parvovirus (CPV) [1] and coronavirus (CCoV) [2], [3], although other agents, such as canine adenovirus (CAdV) type 1 [4], canine distemper virus (CDV) [5], rotaviruses [6], reoviruses [7], and caliciviruses [8], have been associated with enteric disease in dogs. In recent years, novel viruses have been discovered from dogs with enteritis, namely noroviruses [9], sapoviruses [10], astroviruses [11], and kobuviruses [10], [12].

More recently, a dog circovirus (DogCV) was detected in dogs with vasculitis and/or hemorrhagic diarrhoea in the US (13). Circoviruses (family *Circoviridae*, genus *Circovirus*) are non-enveloped, spherical viruses with a small monomeric single-strand circular DNA genome of about 2 kb in length. According to the most recent release of the Universal Virus Database of the International Committee on Taxonomy of Viruses, the genus *Circovirus* consists of eleven recognized species, including Porcine circovirus 1 (PCV-1), Porcine circovirus 2 (PCV-2), Canary circovirus (CaCV), Beak and feather disease virus (BFDV), and other viruses of domestic and wild birds (http://ictvdb.bio-mirror.cn/Ictv/fs_circo.htm). Porcine and avian circovirus infections are characterized by clinical courses that may vary from asymptomatic infections to lethal disease [14].

Two independent studies have shown that, similar to other animal circoviruses, DogCV possesses an ambisense genomic organization with 2 major inversely arranged ORFs encoding for the replicase and capsid proteins, respectively [13], [15]. The canine virus, firstly detected in serum samples [15], was later recognized as causative agent of necrotizing vasculitis and granulomatous lymphadenitis [13].

The aim of this paper is to report the detection and molecular characterisation of DogCV in dogs with acute gastroenteritis in Italy. The full-length genome of the Italian prototype strain was determined and analyzed in comparison with American strains and other circoviruses.

## Materials and Methods

### Ethics statement

The study did not involve any animal experiment. Tissue samples were collected only from dead animals for laboratory analyses, avoiding unnecessary pain and suffering of the animals. The dog owner gave his written consent for necropsy and sample collection.

### Clinical outbreak, post-mortem and sample collection

In June 2013, an outbreak of acute gastroenteritis occurred in a client-owned litter of dachshunds in Apulia, Southern Italy. The six 5–6-months old animals had completed the first-year vaccination protocol against CPV, CDV, CAdVs and *Leptospira* spp. The clinical signs in the dogs were severe, with hemorrhagic diarrhoea, vomiting and death of 2 animals after one week of illness. The other dogs completely recovered within 12–15 days after the onset of clinical signs. One dog carcass was frozen at −20°C after three days of storage at +4°C and sent to our Department for necropsy and laboratory investigations only after three months. Samples from the other dead dog and from the surviving animals were not available for additional analyses, as they were not collected timely.

At post-mortem examination, the dog displayed hepatitis and haemorrhagic enteritis with involvement of the mesenteric lymph nodes that appeared congested and haemorrhagic.

Samples from the liver and intestine of the dog were collected and homogenized in 1 ml viral transport medium consisting of Dulbecco's modified Eagle's medium (DMEM) supplemented with 5% fetal calf serum (FCS), 1000 IU/ml penicillin, 1000 μg/ml streptomycin and 10 μg/ml amphotericin B. Tissue homogenates were clarified by centrifugation at 2,500×*g* for 10 min.

### Nucleic acid extraction

One-hundred-forty microliters of the supernatants were used for RNA and DNA extraction with the QIAamp *cador* Pathogen Mini Kit (Qiagen S.p.A., Milan, Italy), following the manufacturer's protocol and the nucleic acid templates were stored at –70°C until use.

### Detection of common enteric pathogens

DNA/RNA extracts were screened for enteric pathogens of dogs, including CCoV [16], [17], CPV [18], [19], CAdV types 1 and 2 [20], CDV [21], reoviruses [22], rotaviruses [23], caliciviruses [24], astroviruses [25], canine kobuvirus [26], canine minute virus [27], canid herpesvirus type 1 [28]. In the PCR assays, the samples were considered positive if amplicons of the expected size were visualized after gel electrophoresis and staining with ethidium bromide. In the real-time PCR assays, the samples were considered positive if the amplification curves were higher than the threshold line generated by the software on the basis of the background fluorescence.

Standardized procedures were carried out for in vitro isolation of bacteria commonly associated with enteritis. The samples were plated out on 5% sheep blood agar and cultured aerobically at 37°C for 24 h for detection of aerobic pathogens. Bacteriological investigations were carried out by standard biochemical procedures and analytical profile index (API, BioMérieux Italia S.p.A., Rome, Italy). Intestinal parasites were searched for in the faeces or intestinal contents using the zinc sulphate flotation. The Ziehl Nielsen staining was performed on the stools and intestinal sections for identification of *Cryptosporidium* spp.

### Molecular detection of DogCV

All the nucleic acid extracts were subjected to a real-time PCR assay specific for DogCV [13], with minor modifications. Briefly, real-time PCR was performed on a 7500 Real-time PCR System (Applied Biosystems, Foster City CA) with iTaq Supermix added with ROX (Bio-Rad Laboratories Srl, Milan, Italy). The reaction mixture (25 µl) contained 12,5 µl of iTaq Supermix, primers DogCV-forward and DogCV-reverse [13] at a concentration of 600 nmol l^−1^, probe DogCV-probe [13] at a concentration of 200 nmol l^−1^, and 10 µl of template or plasmid DNA. The thermal cycling consisted of activation of iTaq DNA polymerase at 95°C for 10 min and 45 cycles of denaturation at 95°C for 15 s and annealing-extension at 60°C for 1 minute.

### Virus isolation attempts

DogCV positive samples were inoculated into various cell lines that support replication of other canine viruses, i.e. canine fibroma (A-72, ATCC CRL-1542), Madin Darby canine kidney (MDCK, ATCC CCL-34), African green monkey kidney (VERO, ATCC CCL-81), Walter Reed canine cells (WRCC) [29], Crandell feline kidney (CrFK, ATCC CCL-94), and *felis catus* whole foetus (fcwf, ATCC CRL-2787). The liver and intestine were homogenized in D-MEM (10% weight/volume) and 500 µl of the homogenates were used to infect about 1 million cells. The cells were grown in D-MEM supplemented with 10% foetal calf serum (FCS). Since glucosamine has been reported to enhance replication of PCV, the infected cells were also treated with 300 mM glucosamine [30]. When the monolayers were confluent, the medium was removed and the cells were washed twice with FCS-free medium and inoculated with clarified tissue homogenates. After adsorption for 60 min at 37°C, the inoculum was replaced with fresh serum-free medium. Attempts of cultivation were also carried out using freshly-trypsinized cells. The infected cells were monitored daily for the appearance of cytopathic effects (CPE) and, after 5 days of incubation, the inoculated cells were tested for DogCV in real-time PCR. The cells were sub-cultured every 6–8 days for 5 consecutive passages.

### Full-genome sequencing of DogCV strain Bari/411–13

In order to determine the full-length genome of the Italian DogCV prototype strain, a rolling circle amplification (RCA) protocol was performed as previously described [31] using the TempliPhi 100 amplification kit (Amersham Biosciences). Briefly, 1 µl of extracted DNA was mixed with 5 µl of TempliPhi sample buffer supplemented with 450 µM dNTPs, and the mix was incubated at 95 °C for 3 min and subsequently cooled on ice. After adding 5 µl TempliPhi reaction buffer and 0.2 µl TempliPhi enzyme mix, the mixture was incubated at 30 °C for 16 h, and subsequently inactivated at 65 °C for 10 min. For cloning, a total of 30 µl of the RCA product was digested with ApaI and the resulting 2 kb-long fragment was ligated with the ApaI-restricted vector pBluescript II SK(+) (Stratagene) and transformed into XL-1 Blue MRF' Escherichia coli cells (Stratagene). The 2 kb-long insert of the plasmid was sequenced using the universal primers M13 forward and M13 reverse (Invitrogen) at the BaseClear B.V. (Leiden, The Netherlands). A consensus sequence was generated using three clones.

### Sequence analysis

The genome sequence of the DogCV was assembled and analysed using the Genious software package (http://www.geneious.com) and the NCBI's (htttp://www.ncbi.nlm.nih.gov) and EMBL's (http://www.ebi.ac.uk) analysis tools. The complete sequence was deposited in GenBank under accession no. KJ530972.

The nt sequences of the different ORFs were aligned with cognate sequences of reference DogCV strains and of other circoviruses using a translation-based alignment. Phylogenetic and molecular evolutionary analyses were conducted using Mega4.1 Beta [32]. Phylogenetic trees based on the full-length genome and on the nt sequences of the replicase and capsid protein genes were elaborated using both neighbor-joining and parsimony methods, supplying a statistical support with bootstrapping over 1000 replicates. The following reference circovirus strains were used in the phylogenetic analysis: DogCV 214 (JQ821392), UCD1 (NC_020904), UCD2 (KC241984) and UCD3 (KC241983); PCV-1 (AY660574); PCV-2 AUT1 (AY424401), CaCV (AJ301633), BFDV (AF071878), Columbid circovirus (CoCV) (AF252610), Goose circovirus (GoCV) (J30445), Duck circovirus (DuCV) 33753-52 (DQ100076), Raven circovirus (RaCV) strain 4–1131 (DQ146997), Starling circovirus (StCV) (DQ17290), Gull circovirus (GuCV) (DQ845074), Finch circovirus (FiCV) (DQ845075), Cygnus olor circovirus (SwCV) (EU056310), Barbel circovirus (Barbel CV) (GU799606), Cyclovirus (Cy) NG13 (GQ404856), Silurus glanis circovirus (Catfish CV) (JQ011378), CyCV TN25 (GQ404857), CyCV PK5034 (GQ404845), CyCV PK5006 (GQ404844), CyCV NGChicken8 (HQ738643). The distantly-related *Gyrovirus* Chicken anemia virus (CAV) (M55918) was used as outgroup.

## Results

### Detection of DogCV as causative agent of enteritis

The liver and gut samples of the dead dog tested positive in the real-time PCR targeting the replicase gene of DogCV, yielding cycle threshold (C*_T_*) values of 26.4 and 24.3, respectively. There was no evidence for additional pathogens in the analyzed samples. All attempts to adapt the virus to the in-vitro growth were unsuccessful, as shown by the absence of CPE and by the progressive increase of C*_T_* values in the serial passages of the inoculated cell cultures. The fourth and fifth passages tested negative by real-time PCR.

### Full-genomic characterization of the Italian DogCV prototype strain

The full-genome sequence of the Italian DogCV prototype strain Bari/411–13 was determined. The genome of strain Bari/411–13 was of 2,063 nt in length, like all the other DogCVs, with the only exception of strain 214, that is 2-nt shorter. In contrast, PCVs have a genome length of 1,955 nt.

The viral genome displayed the same organization as DogCVs described previously and PCVs, with 2 open readings frames (ORFs), on complementary strands in opposite orientation, which encode for the viral replicase and capsid protein, respectively. Similar to other animal circoviruses, the genome of strain Bari/411–13 contained two intergenic noncoding regions (203 and 135 nt in length) located between the start and stop codons, respectively, of the replicase and capsid protein genes. The 5′-intergenic region contains a thermodynamically stable stem-loop for initiation of rolling-circle replication and a conserved nonanucleotide motif, TAGTATTAC [13], [15]. On the bases of the similarity with other circoviruses, the origin of replication site for strain Bari/411–13 was predicted to consist of a palindromic sequence that includes dodecanucleotide pairs in the stem, whereas the loop is formed by the decanucleotide motif CATAGTATTA. Interestingly, a 150-nt stretch within the 3′-intergenic sequence showed ≈91% nt identity against a torque teno virus recently detected in wild pine marten (*Martes martes*) [33]. The retention of this conserved nucleotide stretch among members of the families *Circoviridae* and *Anelloviridae*, has been interpreted as the result of an intersection in the evolution of these viruses [15].

The overall nt identity of strain Bari/411–13 in the whole genome to other DogCVs ranged from 84.9% (strain 214) to 98.1% (strain UCD2). Excluding circoviruses from dogs, the closest genetic relatedness was found with CoCV (38.5%) whereas identity to PCVs was as low as 37.0–38.0% (Table 1).

**Table 1:** Nucleotide identities (%) of DogCV Bari/411–13 with reference canine circoviruses in different genomic regions.

Circovirus	GenBank accession number	Full-length genome	Replicase protein gene	Capsid protein gene
DogCV-214	JQ821392	96.1	95.3	96.6
DogCV-UCD1	NC_020904	95.2	96.3	93.6
DogCV-UCD2	KC241984	98.1	97.6	98.0
DogCV-UCD3	KC241983	84.9	82.1	85.6

The replicase protein was 303-aa long and contained several aa motifs that are highly conserved among circoviruses, including those involved in RCA (FTINN, TPHLQ, CSK) and the dNTP binding site GCGKS. There were 36 aa changes in the replicase of DogCVs, and 6 changes were unique to the Italian circovirus (Table 2). In this gene, the identity to other DogCVs was generally high (95.3–97.6% nt), although identity to strain UCD3 was lower (82.1% nt). Excluding the canine viruses, strain Bari/411–13 appeared more related to Finch circovirus and Gull circovirus (50.8% and 50.3% nt identity), while identity to PCVs was below 50% nt (Table 1).

**Table 2:** Substitutions in the replicase and capsid proteins that are unique to strain Bari/411–13 compared with extant DogCVs.

Protein name	Nt position^a^	Protein residue^a^	Bari/411–13	214	UCD1	UCD2	UCD3
Replicase	226	76	I	L	L	L	L
	394	132	S	T	T	T	T
	503–504	168	M	T	N	K	N
	742, 744	248	V	L	L	L	I
	809	270	C	N	N	N	N
	895	299	Y	H	H	H	H
Capsid	1631	100	I	V	V	V	V

a Nt positions and protein residues are referred to the sequences of DogCV strain Bari/411–13 (GenBank accession number KJ530972).

The capsid protein, 270 aa in length, displayed at the N terminus a 30-aa-strectch rich in arginine residues, as observed in other circoviruses. There were 25 aa changes among DogCVs in the capsid protein, with a single mutation being unique to strain Bari/411–13 (Table 2). Again, the highest identity was observed to strain UCD2 (98.0% nt), while identity to strain UCD3 was lower (85.6% nt). The genetic relatedness with cognate sequence of circoviruses of other species was low (below 30% nt identity) (Table 1).

### Phylogeny

**Figure 1:**
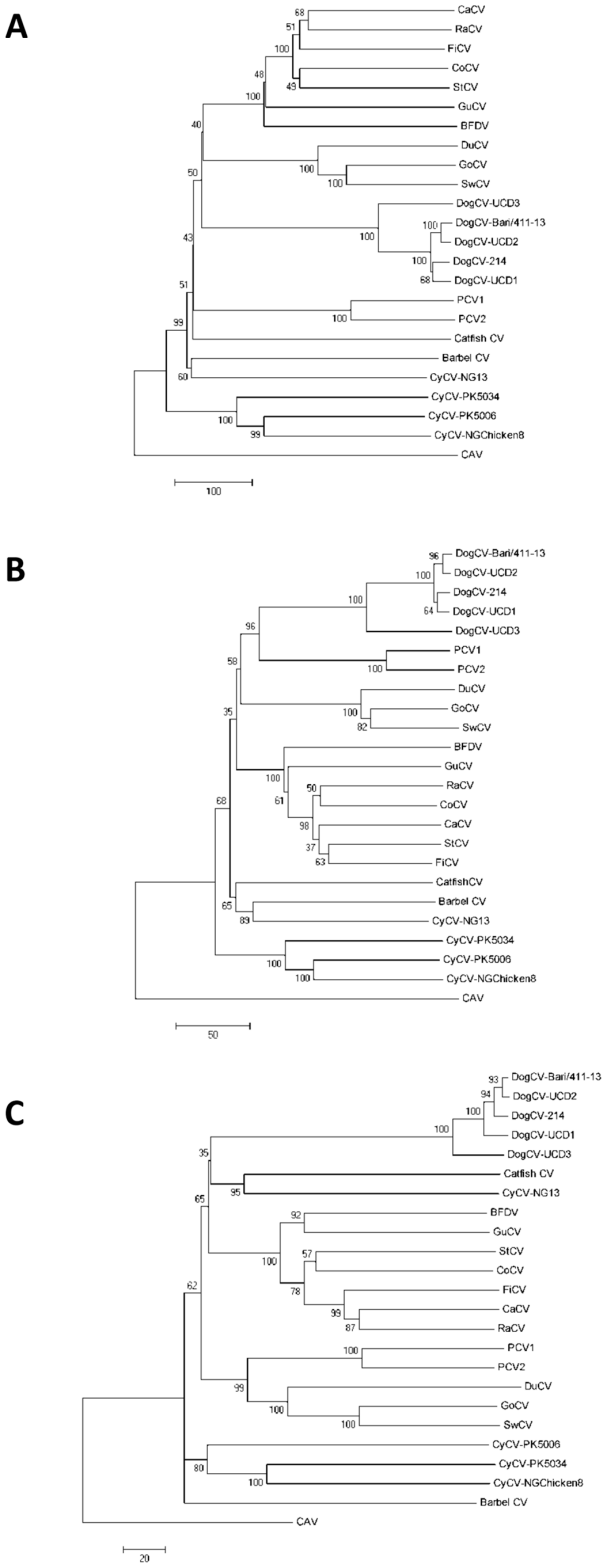
Phylogenetic analysis of DogCV strain Bari/411–13 and other circoviruses. Neighbor-joining trees are based on the full-length genome (**A**), replicase (**B**) and capsid (**C**) protein genes. For phylogenetic tree construction, the reference strains and GenBank accession numbers are as reported in Table 1. The distantly-related *Gyrovirus* Chicken anemia virus (CAV) (M55918) was used as outgroup. A statistical support was provided by bootstrapping over 1,000 replicates. The scale bars indicate the estimated numbers of nucleotide or amino acid substitutions.

In order to investigate the genetic relationship with other CVs, phylogeny was initially inferred using the full-length genome of strain Bari/411–13 and of reference CVs. In the neighbor-joining tree elaborated using the complete genome, strain Bari/411–13 clustered with other DogCVs, whereas PCV-1 and PCV-2 were more distantly related (Fig. 1A). Also, the Italian DogCV was tightly intermingled with other canine circoviruses in the trees constructed on the replicase (Fig. 1B) and capsid protein genes (Fig. 1C). The same tree topologies were obtained in the phylogenetic analysis using the maximum parsimony method (data not shown).

## Discussion

In recent years, a number of novel canine viruses have been discovered. DogCV, first detected in dogs' sera [15] in 2012, was later associated to canine vasculitis and hemorrhage [13]. However, analysis of archival samples has revealed that this novel virus has been circulating in dogs for at least 5 years before being discovered [13]. The data gathered in this study extend the geographical distribution of DogCV to the European continent. In addition, our findings suggest a possible association between DogCV and canine enteritis. Unfortunately, the carcass of the CV-infected dog had been stored frozen for some months before being delivered to our laboratory. Long-term storage at low temperatures hindered the execution of histopathology, and valuable information on tissue localization and alterations of DogCV could not be gathered.

The full-length genome of the Italian prototype strain Bari/411–13 was determined, revealing close genetic relatedness to the American viruses. Virus isolation attempts using different canine cell lines were unsuccessful although the C**_T_** values obtained in real-time PCR amplification were suggestive of high viral titers in the inocula. PCVs have been reported to grow better in the presence of glucosamine, which may help the virus enter the cell nuclei [29], but this procedure was found not to be helpful for DogCV isolation.

In swine, while PCV-1 is almost completely apathogenic, PCV-2 has been proven to cause a systemic disease known as postweaning multisystemic wasting syndrome (PMWS), often in association with other porcine pathogens [14]. However, since PCV-2 is ubiquitous and the disease observed in the field was not successfully reproduced under experimental conditions, several years passed before the scientific community reached a full agreement about its role as causative agent of PMWS [34].

A number of novel circoviruses have been detected in recent years, including human and animal viruses that have been proposed to form the new genus *Cyclovirus* of the family *Circoviridae* [35]. However, very little is currently known about the ability of these novel circoviruses to cause disease in their natural hosts. The present report, if corroborated by further reports, could represent a useful contribution to clarify better the pathogenic potential of DogCV, even if further studies are needed to assess the pathogenic and epidemiological features of this novel canine agent.
